# Supplementary material for: The Role of Fatty Acid Synthase in the Vascular Smooth Muscle Cell to Foam Cell Transition
Source: Cells. 2024 Apr 9;13(8):658. doi: 10.3390/cells13080658 (PMC11048793; doi:10.3390/cells13080658)

Table S1. Antibodies used on this study.

Protein	Company	Catalog number
FASN	Cell Signaling	3180S
CD68	Cell Signaling	86985S
SOAT1	Cell Signaling	35695S
KLF4	R&D Systems	AF3640
ABCA1	Cell Signaling	96292S
Cal 1/2/3	Santa Cruz	SC-136987
TAGLN	Abcam	ab14106
ACTA2	Sigma	A5228

Original blots

The role of Fatty acid synthase in the vascular smooth muscle cell to foam cell transition.

Bethany J. Bogan, Holly C. Williams, Claire M. Holden, Vraj Patel, Giji Joseph, Christopher Fierro, Hugo Sepulveda, W. Robert Taylor, Amir Rezvan, Alejandra San Martin.

Figure 3: (HASMCs +/- Cholesterol)

FASN

273 kDa

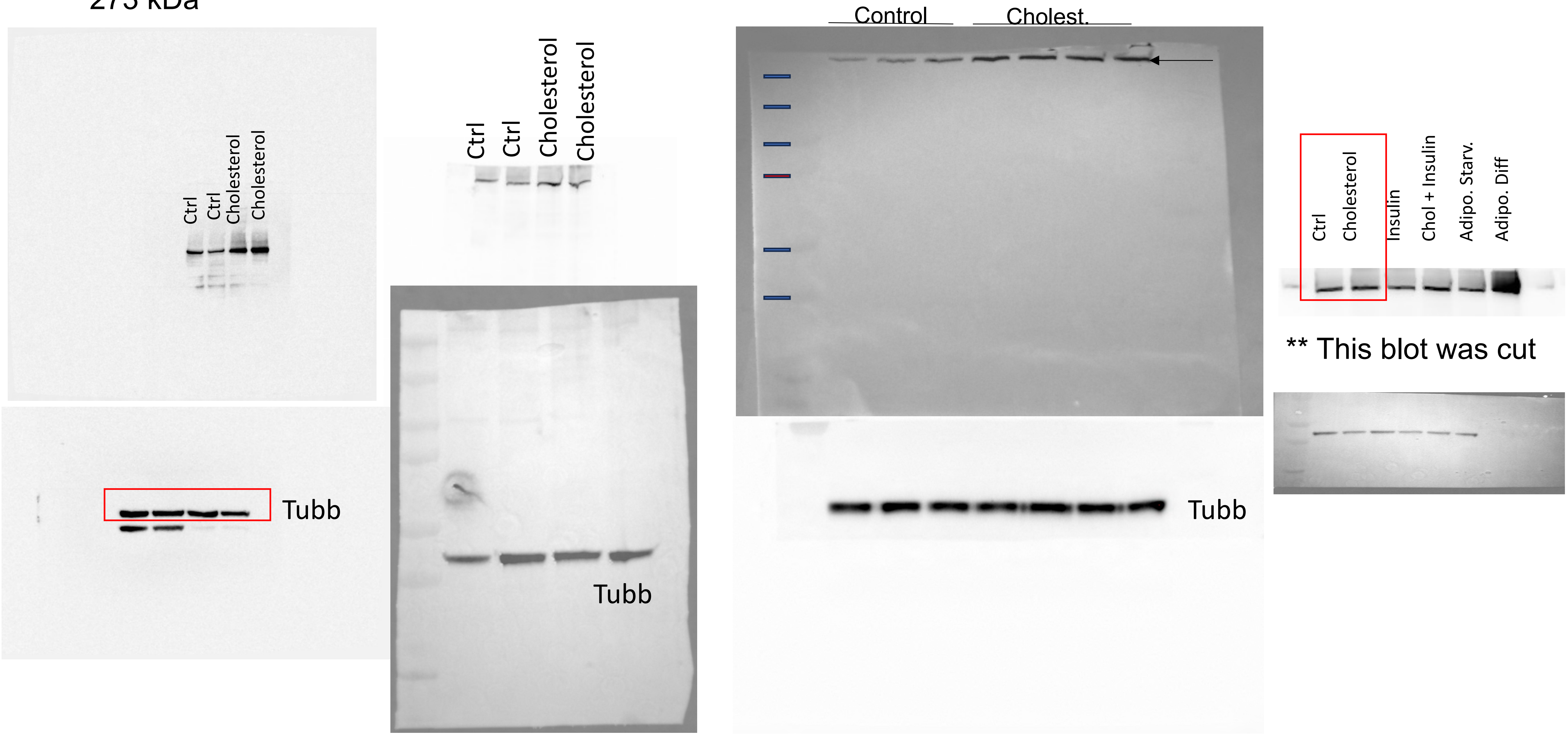

** This blot was cut

Figure 3: (HASMCs +/- Cholesterol)

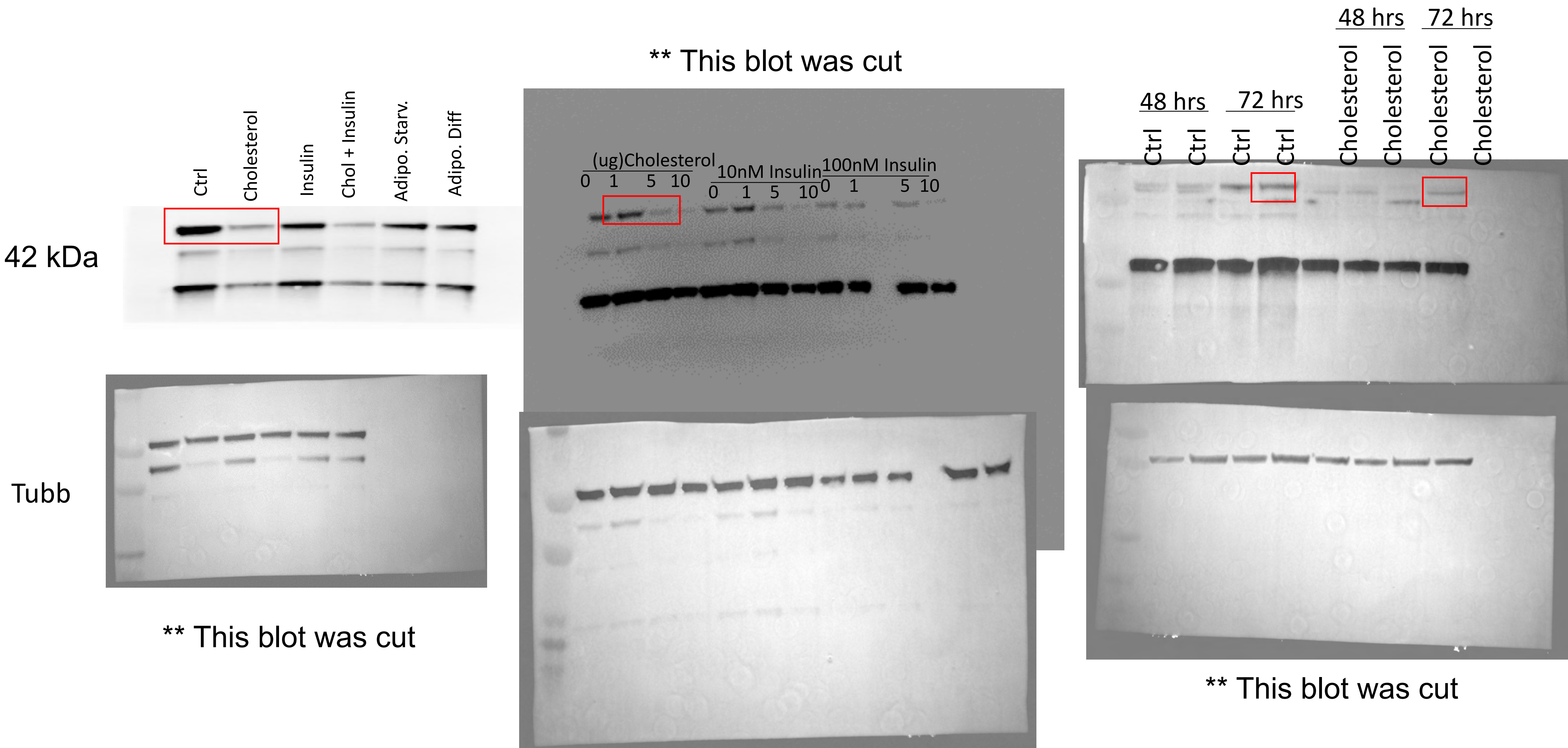

** This blot was cut

** This blot was cut

** This blot was cut

Figure 3: (HASMCs +/- Cholesterol)

SM22

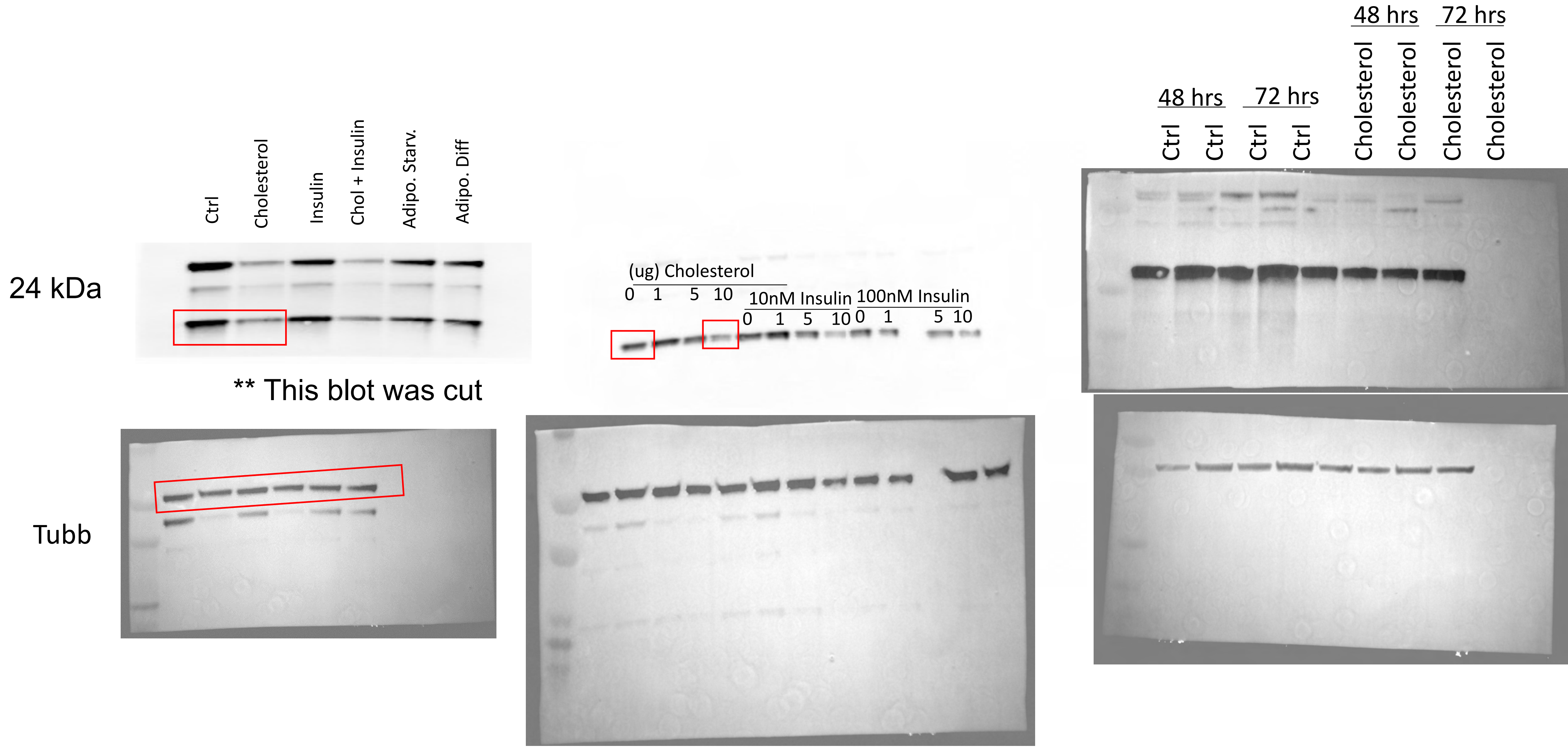

Figure 3: (HASMCs +/- Cholesterol)

KLF4 53 kDa

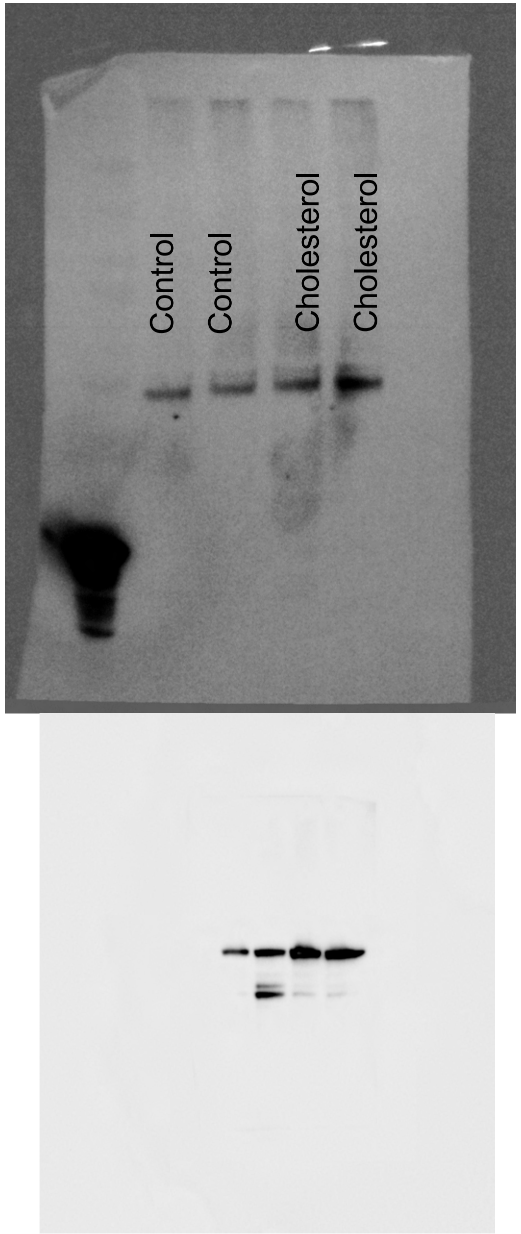

Tubb

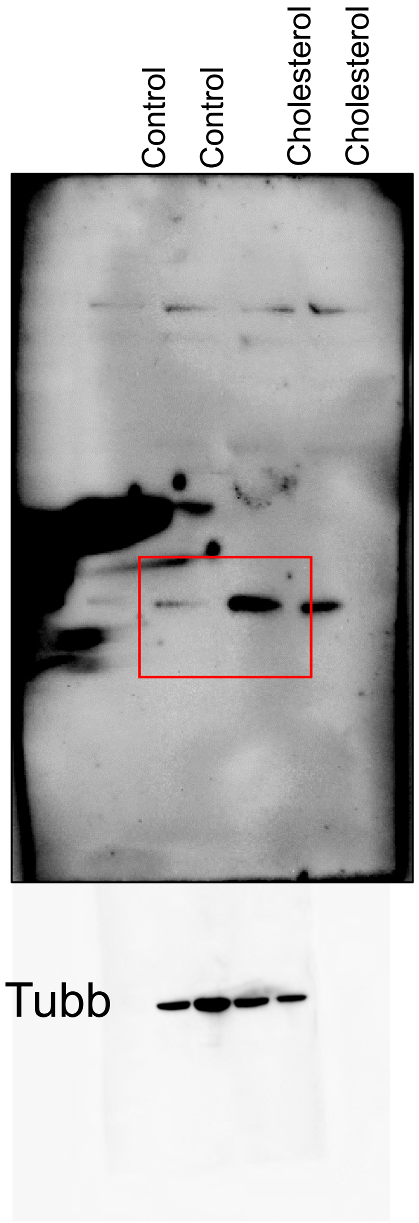

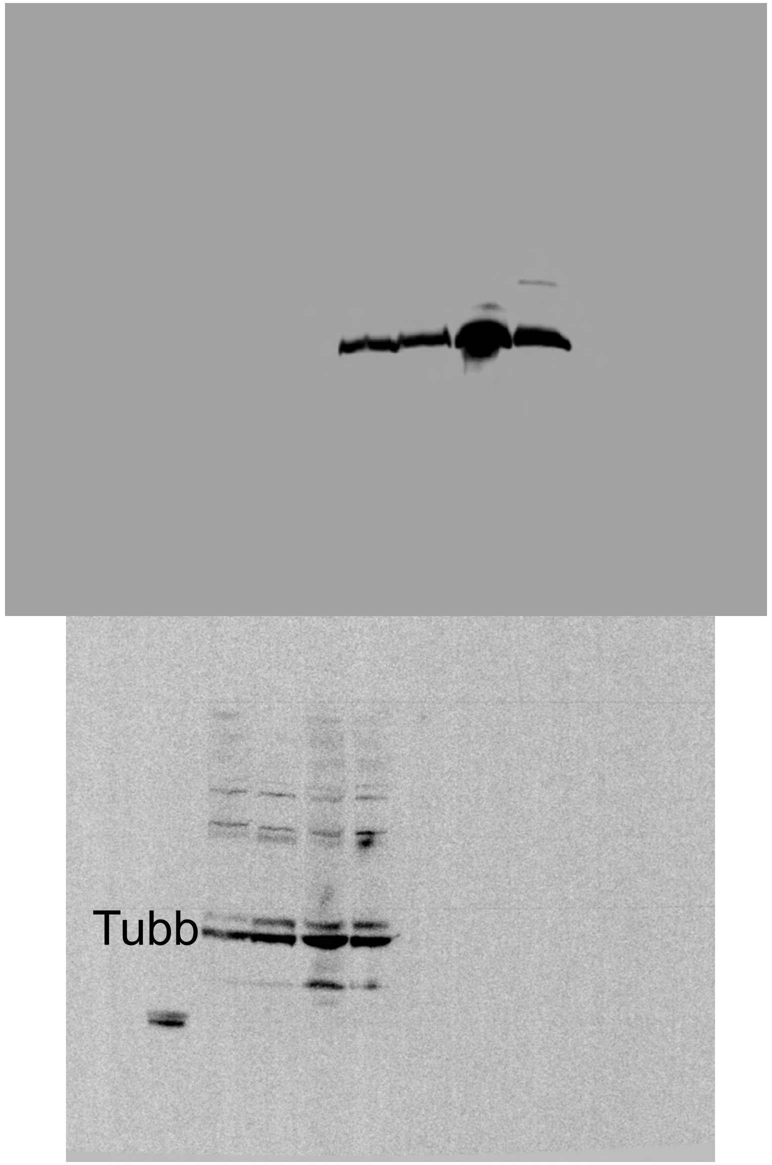

CNN123

Figure 3: (HASMCs +/- Cholesterol)

33-36 kDa

Tubb

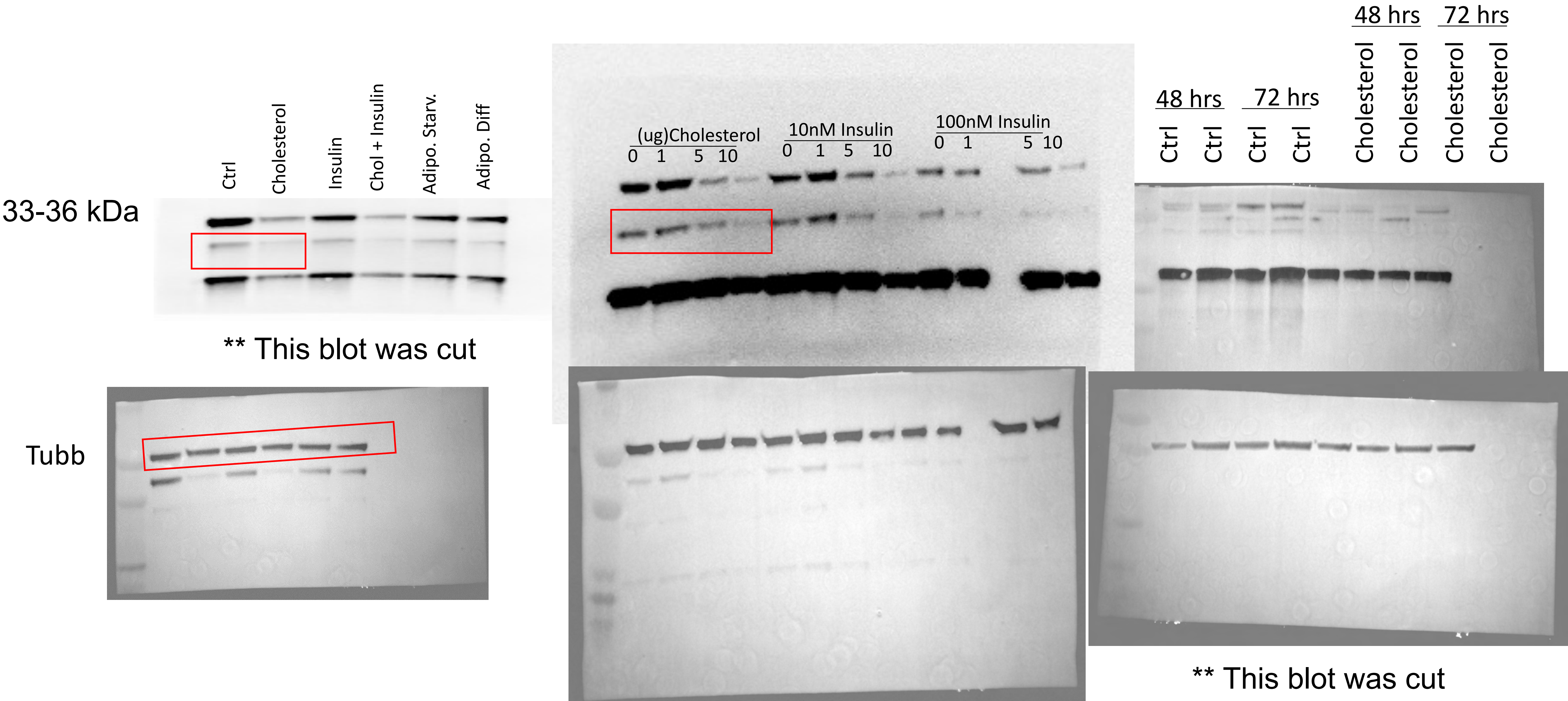

Figure 3: (HASMCs +/- Cholesterol)

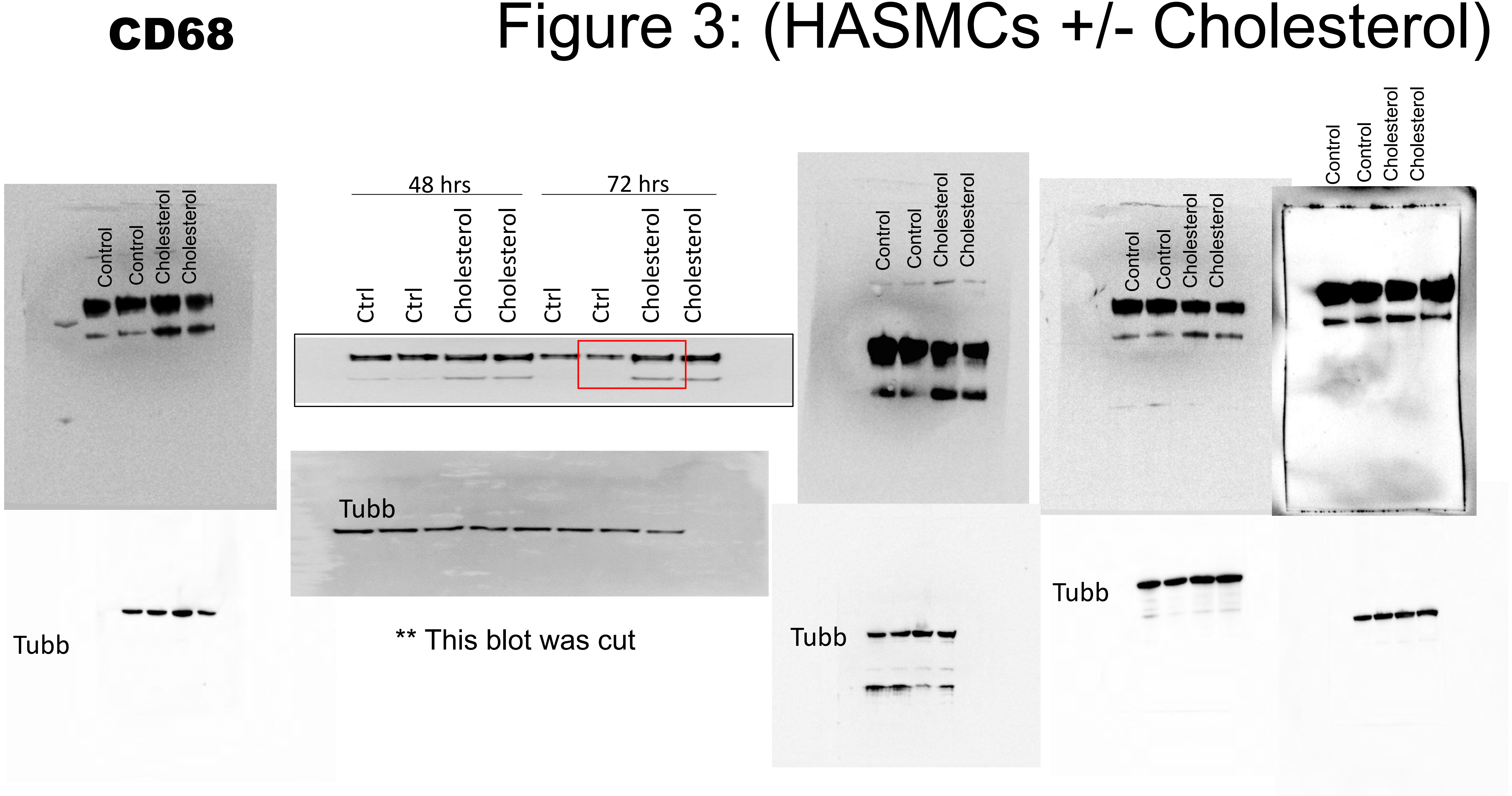

Figure 4: (siFASN +/- Cholesterol)

CD68 110 and 78 kDa

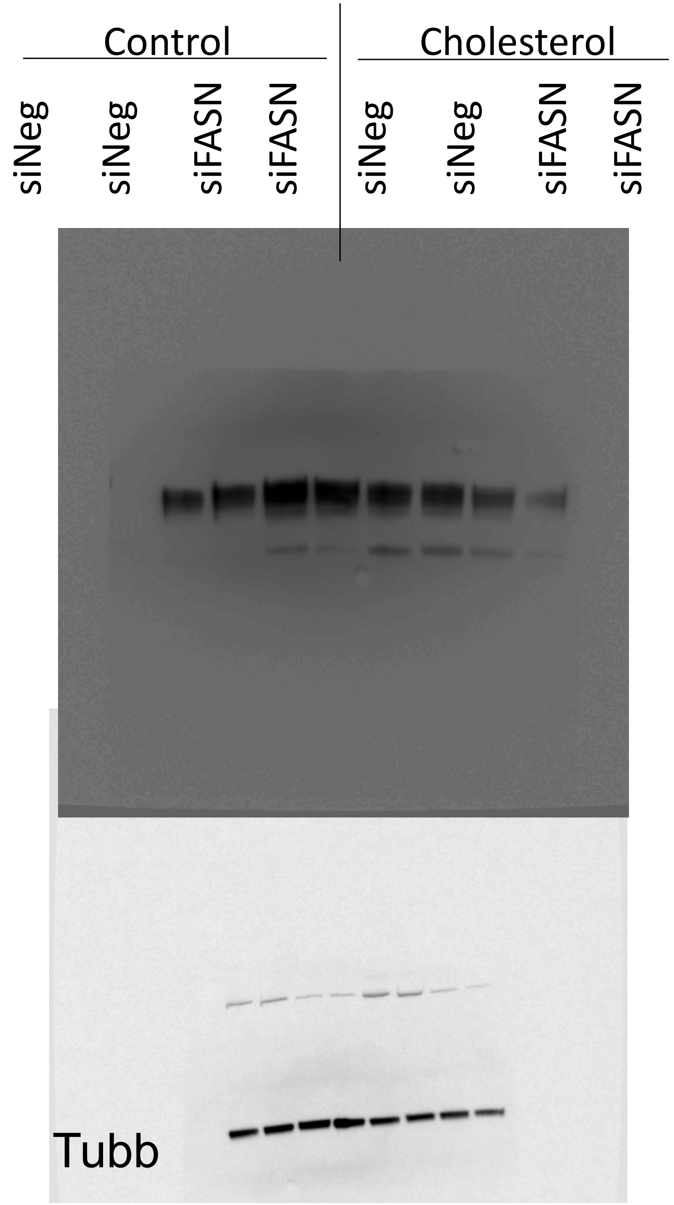

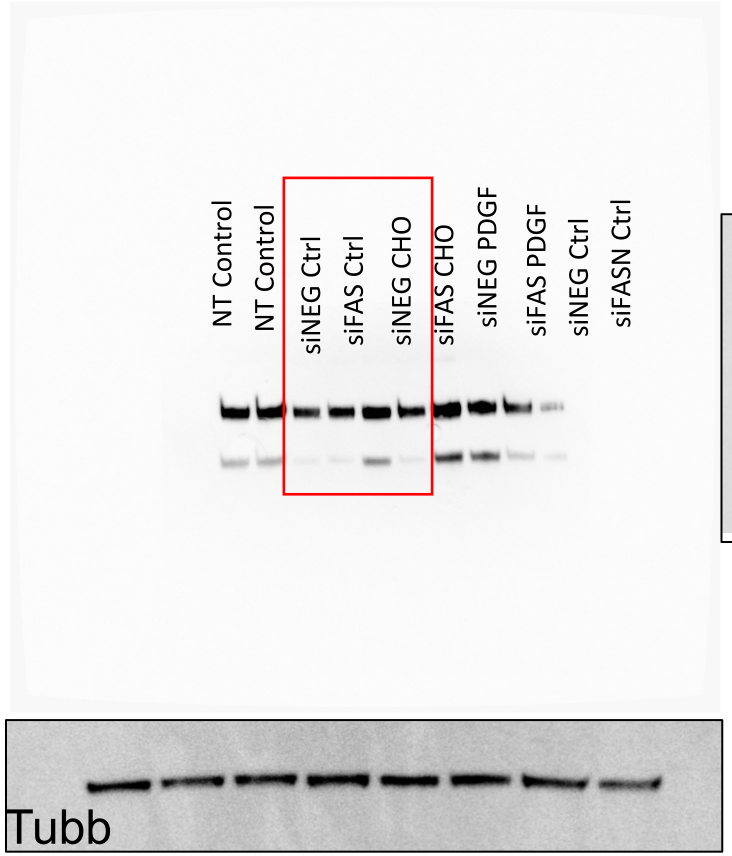

** This membrane was cut during tubulin exposure

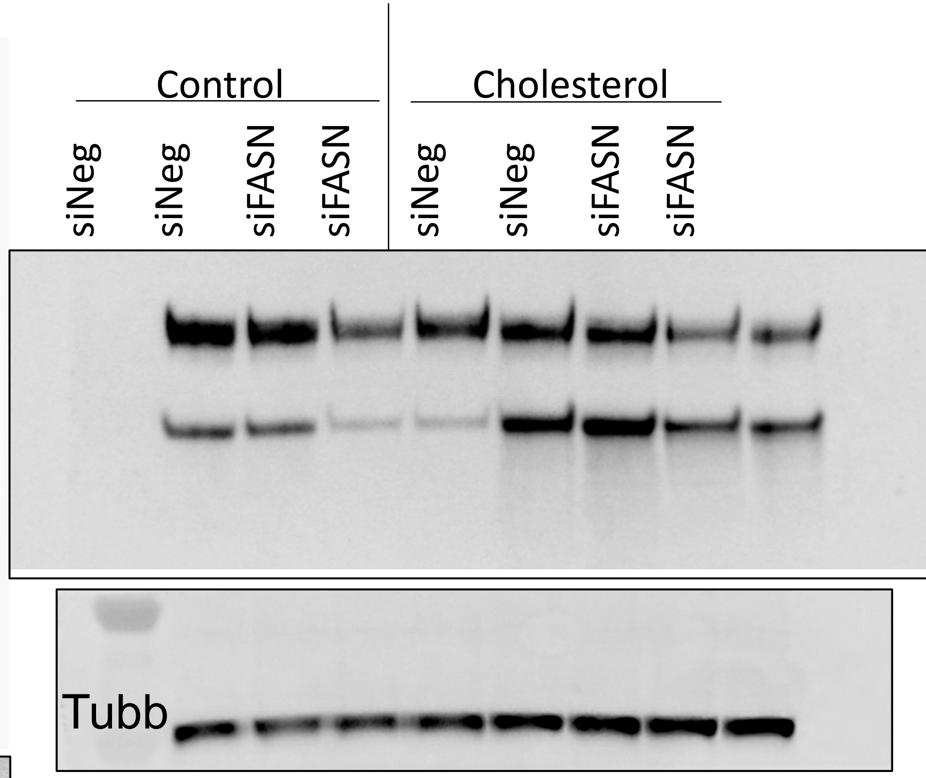

** This membrane was cut

Figure 4: (siFASN+/- Cholesterol)

KLF4

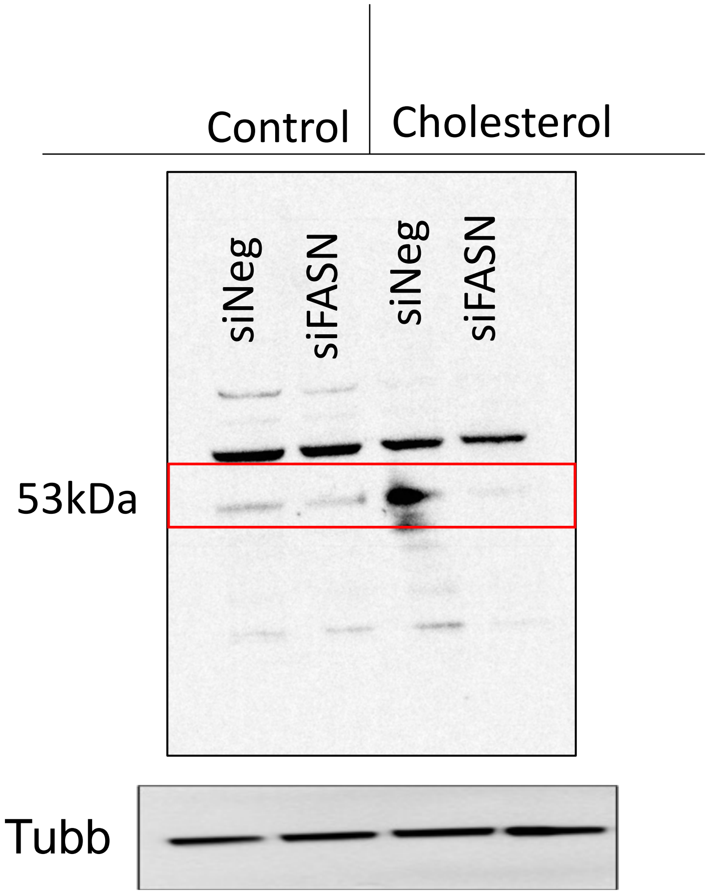

** This membrane was cut during tubulin exposure

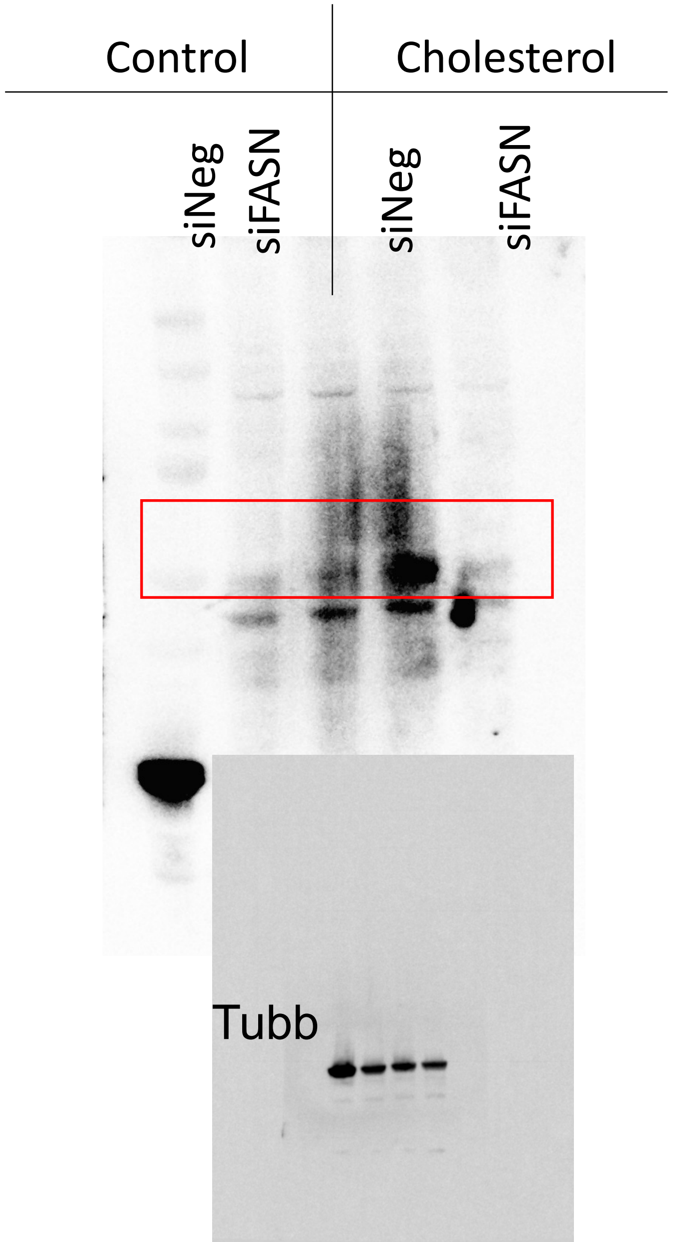

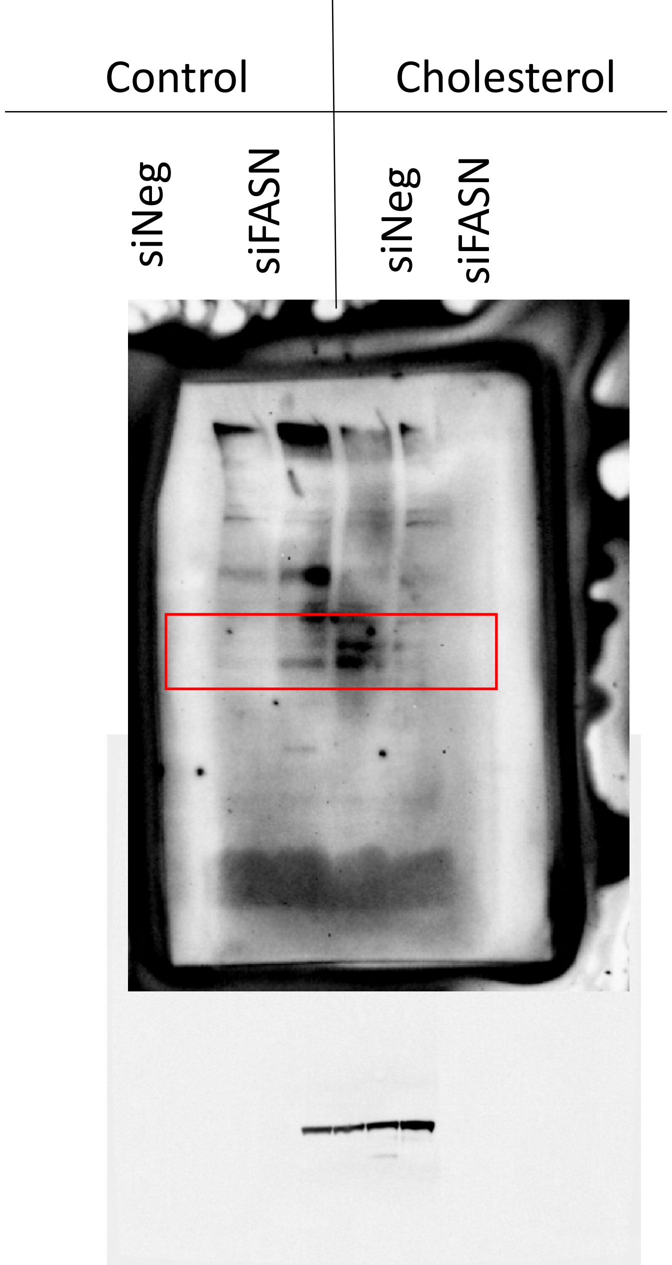

ABCA1

Figure 7: (HASMCs +/- Cholesterol)

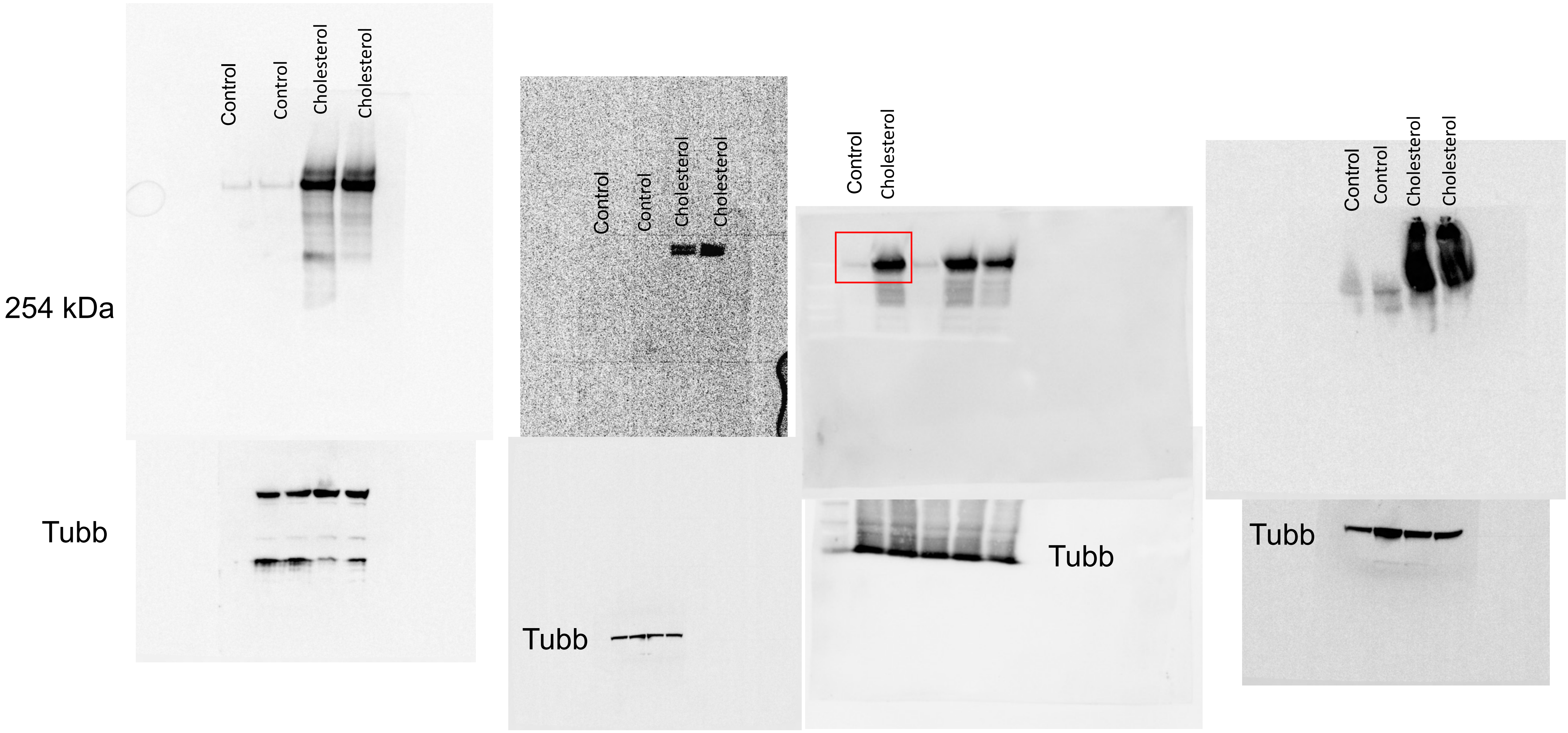

Figure 7: (HASMCs +/- Cholesterol)

SOAT1

45 kDa

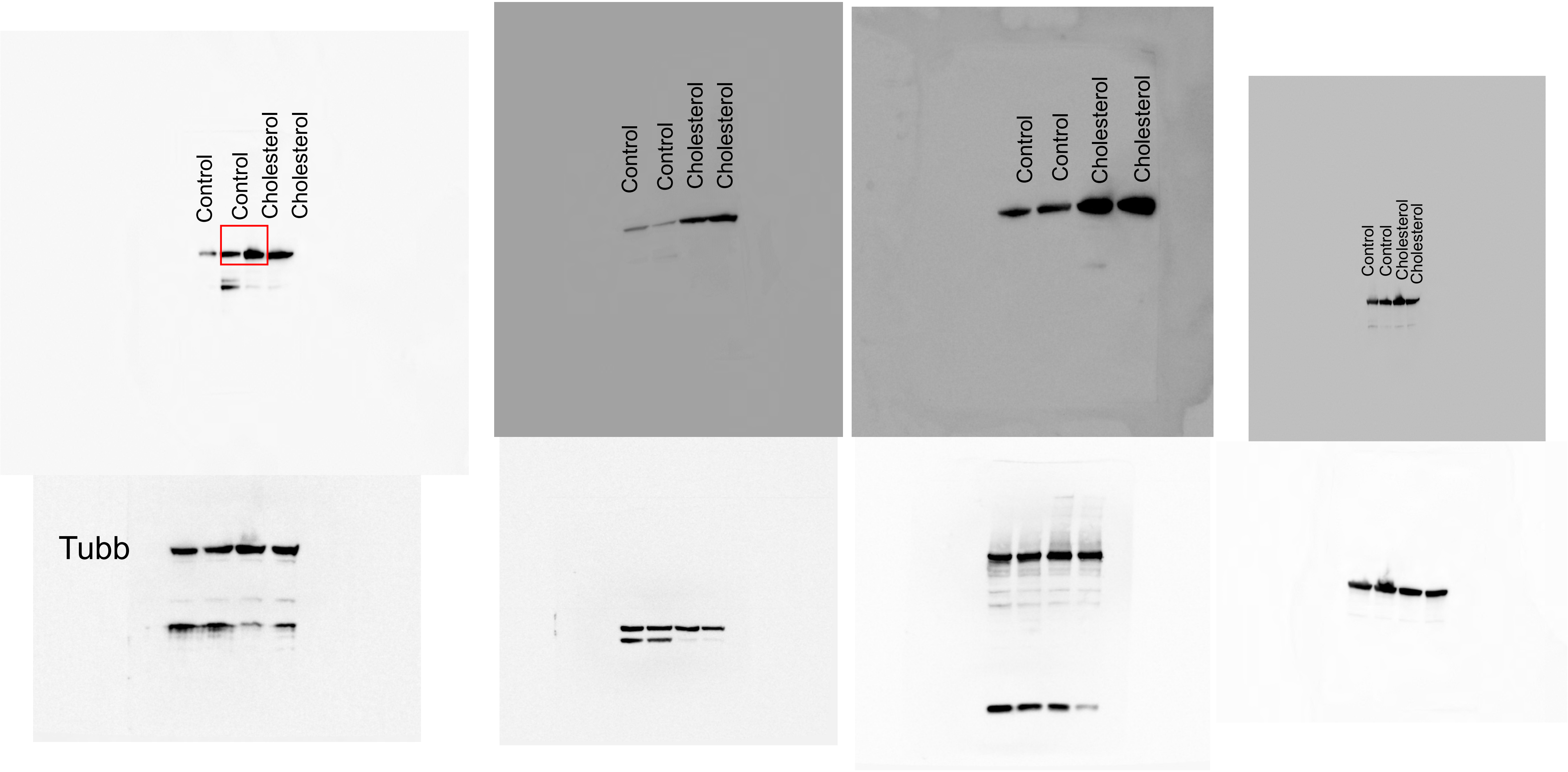

ABCA1, 3-8% gel

Figure 7: (siFASN +/- Cholesterol)

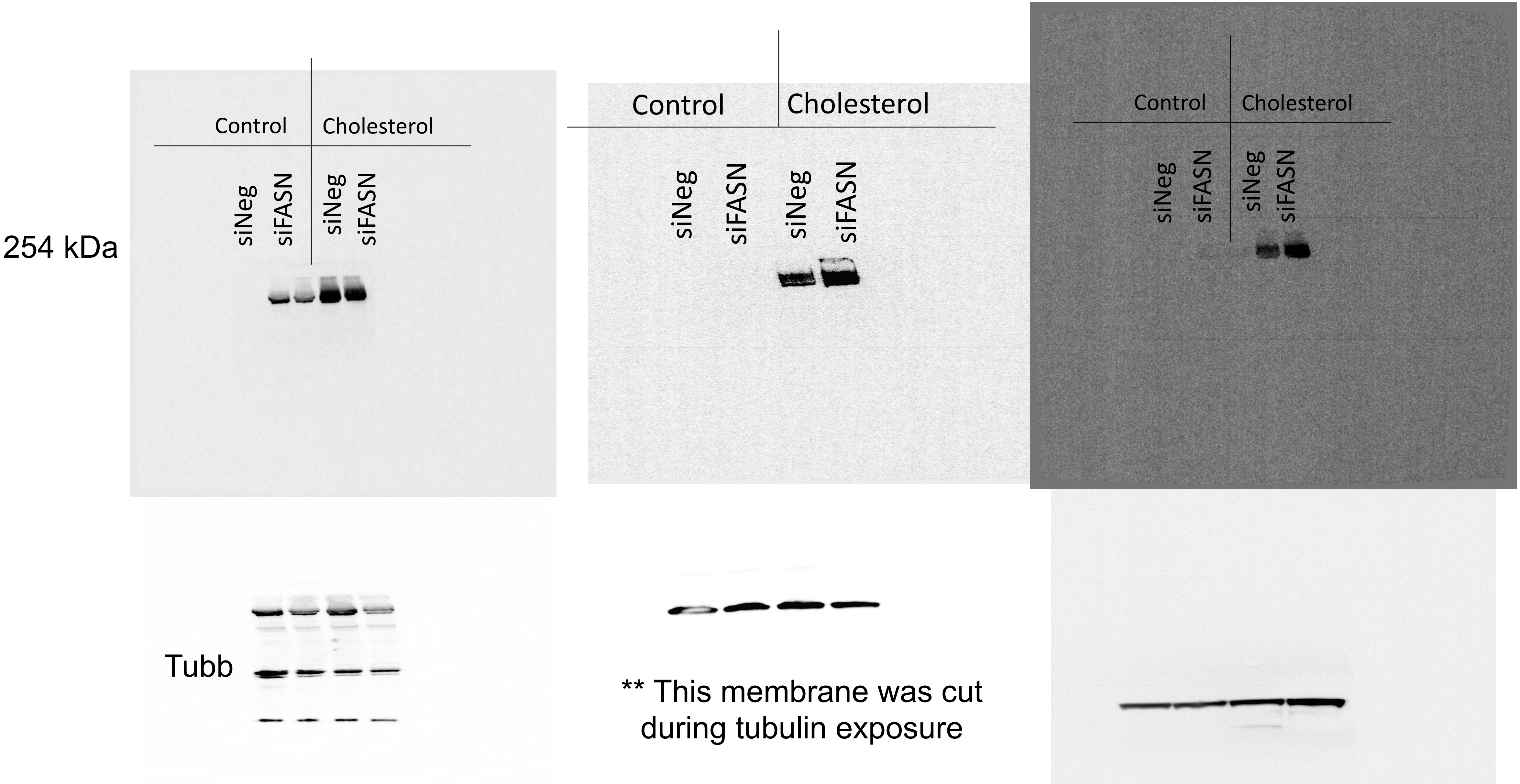

SOAT1

Figure 7: (siFASN +/- Cholesterol)

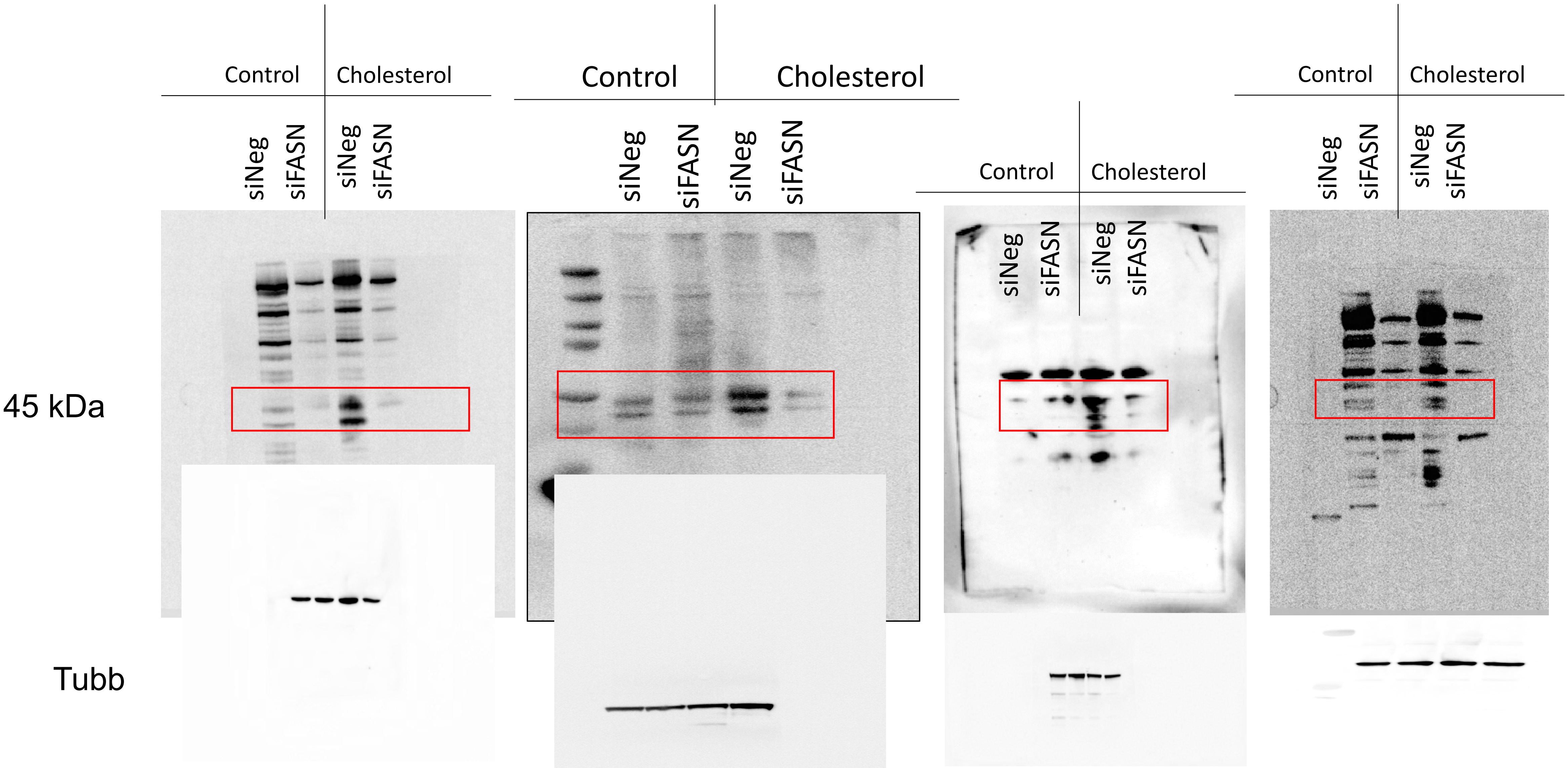

Figure 8:siKLF4 +/- Cholesterol

ABCA1

45 kDa

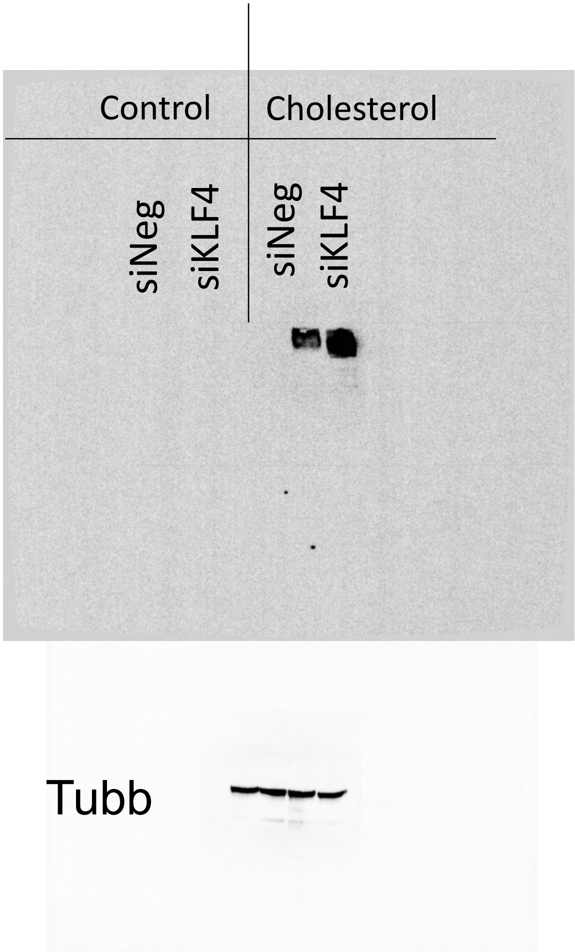

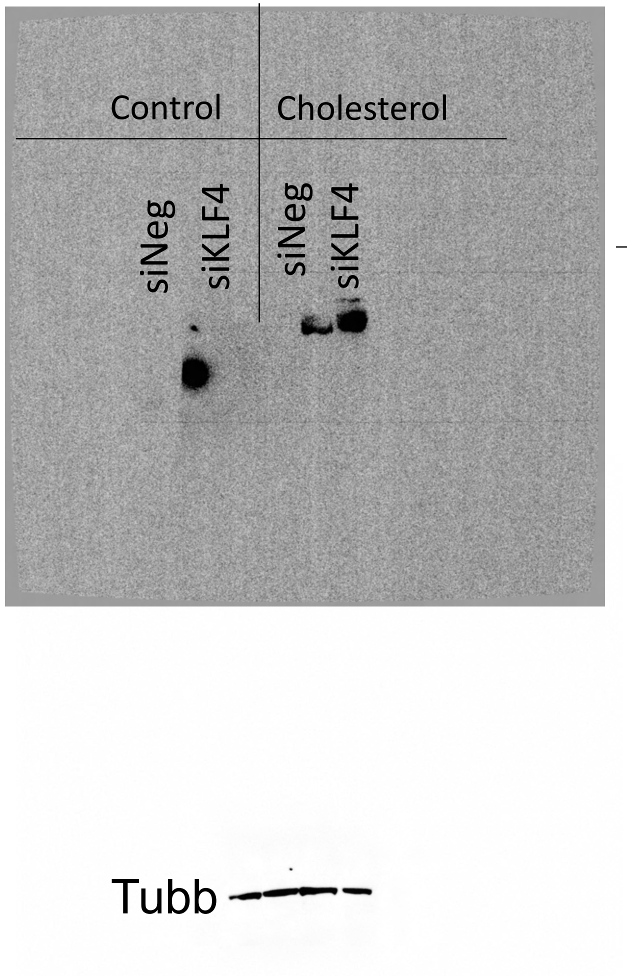

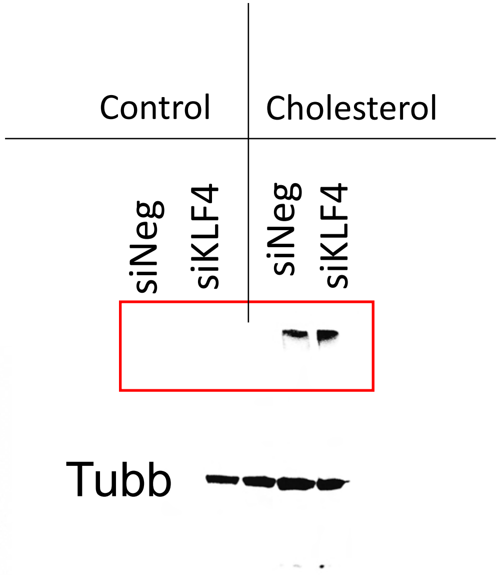

Figure 8: siKLF4 +/- Cholesterol

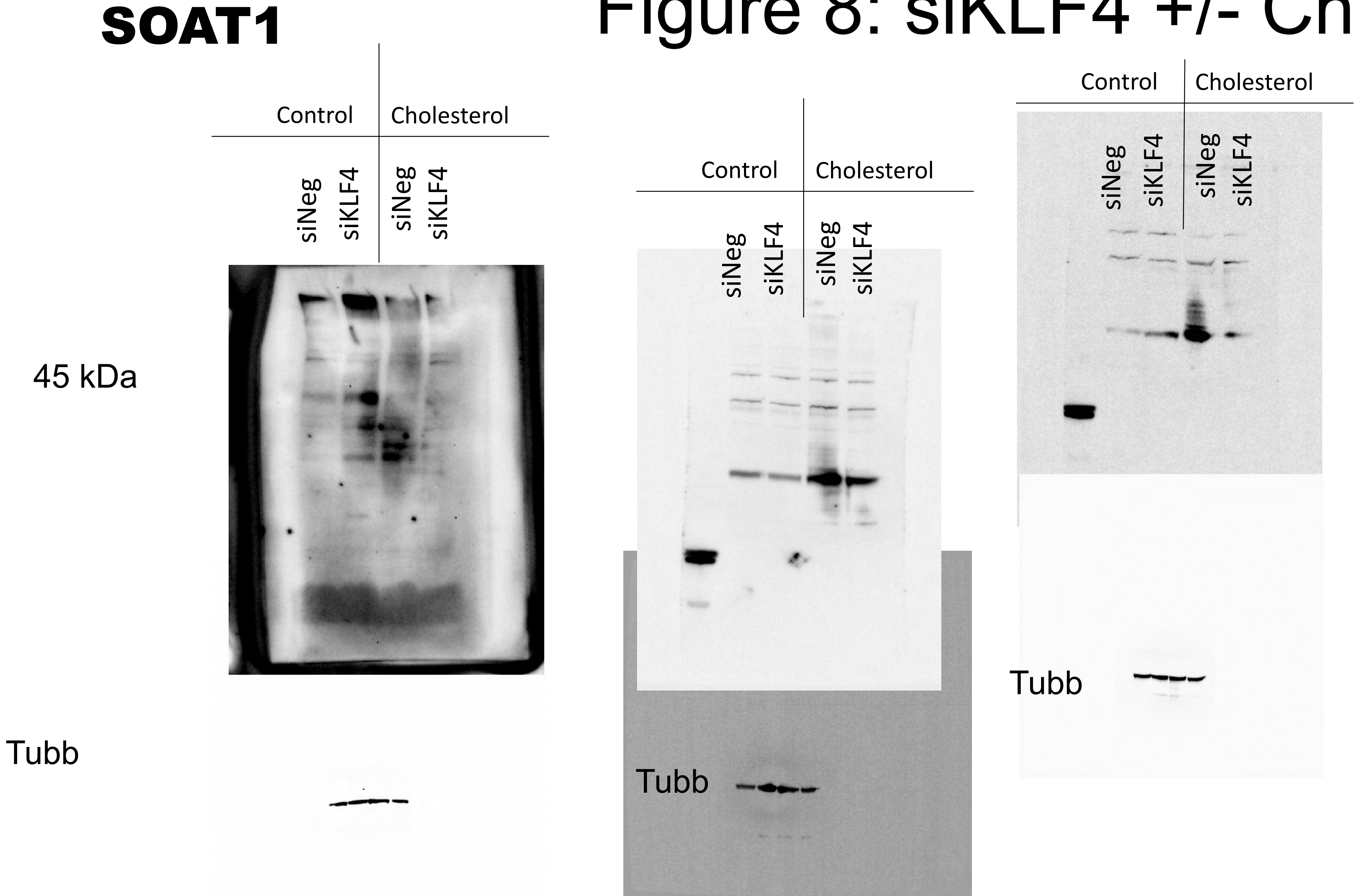

Supplement: Supplementary file 1 [file cells-13-00658-s001.zip › cells-2877597-supplementary.pdf]
